# Growth dynamics of transversal body dimensions and proportions, with related clinical determinants in children with X-linked hypophosphatemia treated with phosphate supplements and active vitamin D

**DOI:** 10.1007/s00467-025-06841-y

**Published:** 2025-06-10

**Authors:** Laura Celine Brieger, Stephan Przygodda, Alina Verena Bohlen, Mirko Rehberg, Martin Konrad, Karl Peter Schlingmann, Olaf Hiort, Dorothee Schmidt, Ulrike John-Kroegel, Elke Wuehl, Markus Josef Kemper, Ute Derichs, Ludwig Patzer, Norbert Albers, Desiree Dunstheimer, Sabine Heger, Karina Grohmann-Held, Carmen Schroeder, Norbert Jorch, Elmar Schmid, Hagen Staude, Marcus Weitz, Clemens Freiberg, Angela Huebner, Anke Heitmeyer-Pyper, Giuseppina Sparta, Carl-Joachim Partsch, Michaela Marx, Christof Land, Inka Baus, Frauke Wilkening, Kristina Moeller, Gunter Simic-Schleicher, Susann Empting, Oliver Metzing, Verena Wagner, Martin Holder, Mislav Stjepan Žebec, Dirk Schnabel, Dieter Haffner, Miroslav Zivicnjak, on behalf of, the German Society for Pediatric Nephrology (GPN), the German Society for Pediatric, Adolescent Endocrinology, Diabetology (DGKED)

**Affiliations:** 1 Department of Pediatric Kidney, Liver and Metabolic and Neurological Diseases, Hannover Medical School, Hannover, Germany; 2 Department of Pediatrics, Faculty of Medicineand, University Hospital Cologne, University of Cologne, Cologne, Germany; 3 Department of General Pediatrics, Pediatric Nephrology, University Children’s Hospital, Münster, Germany; 4 Division of Pediatric Endocrinology and Diabetes, Department of Pediatrics and Adolescent Medicine, University of Lübeck, Lübeck, Germany; 5 Department of Pediatric Nephrology, University Children’s Hospital, Jena, Germany; 6 Center for Pediatric and Adolescent Medicine, Division of Pediatric Nephrology, Heidelberg University, Heidelberg, Germany; 7 Asklepios Children’s Hospital Hamburg-Heidberg, Hamburg, Germany; 8 University Children’s Hospital, Mainz, Germany; 9 Elisabeth and St, Barbara Children’s Hospital, Halle/Saale, Germany; 10 Christliches Kinderhospital Osnabrück, Osnabrück, Germany; 11 Department of Paediatric Endocrinologyand, Diabetology University Hospital of Augsburg, Augsburg, Germany; 12 Kinderkrankenhaus Auf Der Bult, Hannover, Germany; 13 University Children’s Hospital Greifswald, Greifswald, Germany; 14 University Children’s Hospital, Evangelisches Klinikum Bethel, Bielefeld, Germany; 15 Clinic for Pediatric Nephrology Hirschaid, Hirschaid, Germany; 16 University Children’s Hospital Rostock, Rostock, Germany; 17 Department of General Pediatrics and Hematology/Oncology, University Children’s Hospital Tübingen, Tübingen, Germany; 18 Department of Pediatrics and Adolescent Medicine, University Medical Center Göttingen, Göttingen, Germany; 19 Department of Pediatrics, Faculty of Medicineand, University Hospital Carl Gustav Carus, Technische Universität Dresden, Dresden, Germany; 20 Diessen Am Ammersee, Ammersee Ärzte Diessen, Ammersee, Germany; 21 Division of Pediatric Nephrology, University Children’s Hospital Zurich, Zurich, Switzerland; 22 Center for Hormonal and Metabolic Diseases, Reproductive Medicine and Prenatal Medicine, Endokrinologikum Hamburg, Hamburg, Germany; 23 Pediatric Endocrinology, Children’s Hospital Erlangen, Erlangen, Germany; 24 Child and Adolescent Medicine, Gauting, Germany; 25 University MVZ Kiel, Schleswig-Holstein University Hospital, Kiel, Germany; 26 Helios Children’s Hospital Schwerin, Schwerin, Germany; 27 Department of Pediatrics and Adolescent Medicine, Eltern-Kind-Zentrum Prof. Hess, Klinikum Bremen Mitte, Pediatric Nephrology, Bremen, Germany; 28 Klinikum Bremen-Nord, Bremen, Germany; 29 Department of Paediatric Endocrinology and Diabetology, University Children’s Hospital Magdeburg, Magdeburg, Germany; 30 Department of Pediatric Endocrinology, University Children’s Hospital, Jena, Germany; 31 Pediatric Practice Rostock - Endocrinology and Diabetology, Rostock, Germany; 32 Pediatric Diabetology and Endocrinology, Klinikum Stuttgart, Children’s Hospital, OlgahospitalStuttgart, Germany; 33 Institute for Anthropological Research, Zagreb, Croatia; 34 Center for Chronically Sick Children, Pediatric Endocrinology, Charité-Universitätsmedizin Berlin, Berlin, Germany

**Keywords:** XLH, Body disproportion, Transversal body dimension, Frame index, Alkaline phosphatase, Phosphate, Rickets

## Abstract

**Background:**

Children with X-linked hypophosphatemia (XLH) present with rickets, leg deformities, and growth failure. Bone stability depends on balanced bone growth in both length and width. Data on body proportions, including transverse body dimensions, in children with XLH treated with phosphate supplements and active vitamin D are lacking.

**Methods:**

Six major transverse body dimensions of the trunk and extremities, and the frame index (FI), i.e., ratio between bicondylar humerus diameter and height, were measured annually along with clinical characteristics in 109 pediatric patients with XLH, all on supplementation therapy, participating in a prospective multicenter observational study conducted since 1998. Associations between anthropometric and clinical parameters were investigated using linear mixed-effects models.

**Results:**

Children with XLH exhibited persistent hypophosphatemia and elevated alkaline phosphatase *z* scores despite supplementation treatment. This was associated with disproportionate transversal skeletal growth, which was most pronounced during adolescence (13–17 years). Bicondylar diameter *z* scores (tubular bone width) and FI progressively increased with age (each *p* < 0.05). In addition, FI was identified as a superior indicator of stunting when compared to other measures of transversal dimensionality across all age groups. In young children (2–6 years), transversal growth was most synchronized and associated most strongly with clinical characteristics.

**Conclusions:**

Our data show disproportionate growth in transversal body dimensions despite supplementation treatment in children with XLH, suggesting compensatory widening of tubular bones as adaptation for mineral loss caused by persisting rickets. The FI can be used as a general indicator of bone health in children with XLH in clinical practice and trials.

**Graphical abstract:**

**Figure:**
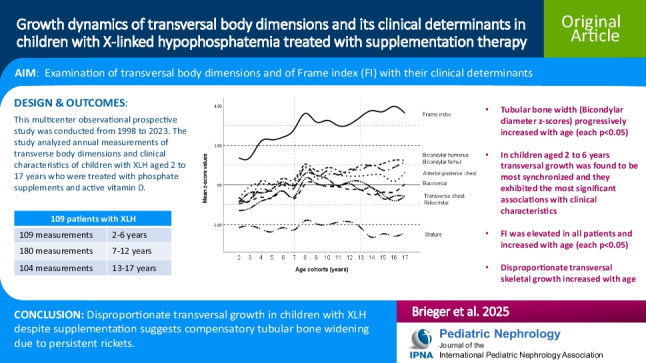
A higher resolution version of the Graphical abstract is available as Supplementary information

**Supplementary Information:**

The online version contains supplementary material available at 10.1007/s00467-025-06841-y.

## Introduction

X-linked hypophosphatemia (XLH) is a rare hereditary disorder with an incidence of 3.9 per 100,000 live births and caused by pathogenic variants of the phosphate regulating endopeptidase homolog, X-linked (PHEX) gene [1]. PHEX malfunction results in increased levels of the phosphaturic hormone fibroblast-growth factor 23 (FGF23) with consecutive renal phosphate wasting, suppressed synthesis of 1,25-dihydroxyvitamin D and hypophosphatemia [2]. The latter causes rickets, osteomalacia, enthesopathy, pseudofractures and tooth abscesses [1, 3]. Treatment with oral phosphate supplements and active vitamin D was shown to only partly correct rickets indicated by persistently elevated alkaline phosphate (ALP) levels, and progressive leg deformities in children with XLH [4–6]. In addition, children on supplementation therapy show progressive disproportionate short stature, characterized by reduced leg growth and preserved trunk growth resulting in an increased sitting height index (SHI), i.e., the ratio between trunk length and total body height [5, 7]. It is unknown if XLH also results in alterations of transversal body dimensions, i.e., of the trunk and tubular bones.

The investigation of transversal body measurements and body indices provides new information, as transversal body dimensions correlate with external and internal factors, e.g., biacromial diameter with state of fitness, and biiliocristal diameter with maturation [8–10]. The frame index (FI) displays a growth disparity pattern between body height and bicondylar humerus breadth and is a useful marker of skeletal strength in the healthy population [11–13].

A low FI is often found in athletes, indicating narrow but dense and strong bones due to high levels of physical activity, which increase bone density and strength. Conversely, a high FI is observed in pathological conditions or due to low levels of physical activity, reflecting reduced functional robustness. This definition is supported by research, including studies by Scheffler et al. and Rietsch et al., which explore the relationship between bone morphology, physical activity, and pathology [11, 14].

Finally, rickets also causes widening of the epiphyseal growth plates and significant widening of the mediolateral femoral width [15, 16].

Therefore, we hypothesized that XLH results in disproportionate growth in the transversal dimensions and body disproportion despite supplementation therapy. The validity of this hypothesis was determined by assessing the FI, as it displays the growth disparity patterns between body height and tubular bone width in the context of bone stability, as bone stability is dependent on balanced bone growth in both length and width.

To this aim, we analyzed detailed anthropometric data and its possible clinical predictors in 109 children with XLH, all on supplementation therapy, undergoing annual assessments in a prospective multicenter observational study in Germany, Austria, and Switzerland initiated in 1998.

## Material and methods

### Study design and patients

In 1998, a prospective multicenter observational study on growth and body proportions in children with XLH was initiated at Charité University Hospital, Berlin. It was extended to Hannover Medical School in 2000, and to the participating centers on behalf of the German Society for Pediatric Nephrology (GPN) and the German Society for Pediatric Endocrinology and Diabetology (DGKED) in 2003 [7]. The diagnosis of patients with XLH aged between 2 and 17 years is made based on family history and/or genetic confirmation, in conjunction with the presence of clinical and/or radiological signs of rickets, in particular reduced height velocity and serum phosphate levels, associated with selective renal phosphate wasting, provided that there is no deficiency of either vitamin D or calcium. A total of 181 patients with XLH were assessed in 43 participating centers in Germany, Austria, and Switzerland. All observed patients received therapy that corresponds to the treatment recommendations currently in place. Historically, the treatment of children primarily entailed the administration of supplemental therapy, and, in cases of severe growth retardation, recombinant human growth hormone (rhGH), resulting in a substantial improvement in linear growth [17]. In 2018, burosumab, an FGF23-neutralizing antibody, was introduced in Europe, and as a result, many patients were switched from purely supplemental therapy to burosumab. Furthermore, patients diagnosed after 2018 were predominantly treated with burosumab in accordance with established guidelines. Here, we focus on anthropometric data obtained in XLH patients treated only with phosphate supplements and active vitamin D, as the follow-up time is still too short to allow for a meaningful analysis in patients who were treated with burosumab. Moreover, treatments with burosumab and rhGH have been shown to improve (bone) growth in pediatric XLH patients, respectively [1, 18]. To exclude a possible bias due to treatment with rhGH or burosumab, we excluded in the present analysis measurements of patients who received rhGH (*N* = 11; 6.1% of the study cohort) or burosumab (*N* = 61; 33.7% of the study cohort), but include the previous data while they were receiving only supplementation therapy [1, 18]. Thus, a total of 109 patients, who were receiving supplemental therapy, were included in the study and underwent 393 annual measurements (3.6 measurements per patient; Table 1), which included anthropometric and clinical assessments, patient history, physical examination, and biochemical parameters for disease monitoring. Patients and/or parents/guardians provided written informed consent and assent for participation in the study. This multicenter prospective observational study was approved by the Ethics Committee of Hannover Medical School (No. 7259) and ethics committees of the individual participating centers, and was performed in accordance with the ethical standards as laid down in the 1964 Declaration of Helsinki and its later amendments.

**Table 1:** Clinical characteristics of 109 children with XLH on supplementation therapy

*Non-repeated measurements*	*Incidence %*	*N*	*p value*
Female sex	63.3	69 of 109	< 0.01
Affected parent with XLH	57.8	63 of 109	< 0.05
Affected mother	76.2	48 of 63	< 0.01
***Non-repeated measurements***^***a***^	***Mean (*± *SD)/median (IQR)***	***N***	
Age at diagnosis, years	2.00 (0.06 to 3.16)	101 of 109	
Age at start of treatment, years	2.03 (0.62 to 3.42)	82 of 109	
Age at menarche, years	12.78 (± *1.45*)	23 of 109	
Father’s height, cm	178.0 (171.0 to 182.0)	107 of 109	
Mother’s height, cm	162.0 (152.0 to 169.0)	107 of 109	
***Repeated measurements***^***b***^	***Estimated marginal mean (95%CI)***	***N***	
Age, years	9.89 (9.19 to 10.59)	393 of 393	
Serum hemoglobin, g/dL	13.30 (13.08 to 13.51)	161 of 393	
Serum phosphate, *z* score	− 3.09 (− 3.39 to − 2.79)	286 of 393	
Serum calcium, *z* score	− 0.21 (− 0.37 to − 0.06)	279 of 393	
Serum ALP, *z* score	2.60 (2.26 to 2.94)	176 of 393	
Serum PTH, *z* score	1.85 (1.55 to 2.15)	271 of 393	
Serum creatinine, µmol/l	40.2 (38.1 to 42.3)	285 of 393	
eGFR, ml/min per 1.73 m^2^	121.4 (116.2 to 126.6)	285 of 393	
Phosphate dosage, mg/kg/day^c^	50.2 (1.5 to 98.9)	393 of 393	
Calcitriol dosage, ng/kg/day^c^	25.6 (14.5 to 36.6)	393 of 393	

^a^Descriptive statistics (non-repeated measurements) are given as mean and standard deviation, or as median and interquartile range (25 th–75 th percentile)

^b^Repeated measurements (estimated marginal means) during the observation period are based on actual measurements or annual average values (AA), repeated measurements with the same individual (evaluated with the linear mixed model, random patients, and age cohorts). Repeated measurements are given as mean and standard deviation or as median and interquartile range (25 th–75 th percentile)

^c^Based on elemental phosphorus

N describes number of either patients (for static patient dependent characteristics) or yearly measurements of overall valid patient cases or valid yearly measurements

*SD*, Standard deviation; *IQR*, interquartile range; *ALP*, alkaline phosphatase; *PTH*, parathyroid hormone; *eGFR*, estimated glomerular filtration rate

### Methods

Annual anthropometric assessments were performed by the same investigator (M.Ž.), with standardized equipment, in accordance with the guidelines of the International Biological Program [7, 19, 20]. Transversal dimensions of the torso (biacromial diameter, transverse chest diameter, anterior–posterior chest diameter, and biiliocristal diameter), as well as measurement of tubular bone width (bicondylar humerus diameter and bicondylar femur diameter) were assessed together with body height. Measurements were further utilized to calculate body indices: body mass index (BMI), sitting height index (SHI), and frame index (FI).

The FI, marker of skeletal robustness, is independent of body fat, unlike BMI [11, 14, 21]. In our study on XLH patients, we chose FI and elbow breadth as anthropometric measures, based on their low correlation with skinfold thickness in healthy individuals [13].

FI was calculated according to the formula:

$$\text{FI}= \frac{\text{elbow breadth }(\text{mm})}{\text{stature }(\text{cm})}\times 100$$

For those anthropometric characteristics, age- and sex-dependent *z* scores were calculated using anthropometric measures from reference data derived from healthy children [19, 22].

At each anthropometric assessment session, clinical data on biochemical characteristics were gathered. Established laboratory methods were utilized to assess serum levels of calcium, phosphate, alkaline phosphatase (ALP), intact parathyroid hormone (PTH), creatinine, and hemoglobin (Hb). Estimated glomerular filtration rate (eGFR) was calculated using the revised Schwartz equation [23]. *Z* scores for ALP, PTH, and phosphate were calculated using age- and sex-specific reference values from the CALIPER [24], as done in a previous publication investigating rickets in XLH patients [5], while calcium *z* scores were calculated using reference values from Kieviet et al. [25]. Presence of nephrocalcinosis was evaluated by kidney ultrasonography. The age at diagnosis and age at menarche were collected from patients’ personal health records or anamnesis. Genetic diagnostics were obtained for 56 of 109 patients, displaying *PHEX* mutations in 80.4% of investigated patients.

### Statistical analysis

Due to the limited number of patients, analysis by singular age (e.g., age 2 = 2.00–2.99 years, 3 = 3.00–3.99 years) was not feasible. Therefore, measurements were clustered into three age groups, which coevally represent biological developmental stages: (A) 2–6 years with 109 total measurements (*N* = 21, representing early childhood); (B) 7–12 years with 180 total measurements (*N* = 44, representing pre-pubertal and early pubertal age); and (C) 13–17 years with 104 total measurements (*N* = 44, representing adolescence). Because of the repeated measures component of the research design, the distribution of measurement data into three age groups may lead to multiple measurements of certain patients falling within and across age groups. Different descriptive statistics were used depending on the related presumptions. For continuous variables, the mean ± SD and/or the median with interquartile range (IQR) is given. For categorical variables, corresponding percentages of overall measurements were provided. For repeated measurement analysis, the estimated marginal mean (EMM) with a 95% confidence interval (CI) was presented. The normality of data distribution was evaluated by using the Shapiro–Wilk test. To compare continuous variables between two groups, the *t* test or Mann–Whitney test were applied, as appropriate. To test the differences in the frequency of subjects’ characteristics measured on a nominal scale, the chi-square test was conducted (MedCalc Software Ltd. Version 22.014). To assess differences in patients’ characteristics gathered through repeated measurements across age groups (A: 2–6, B: 7–12, and C: 13–17 years), linear mixed-effects models (MIXED procedure) were employed. Estimates for the intercept were calculated and tested for statistical significance using the Wald *Z* test. The unstructured covariance matrix type (UN) proved to be the most appropriate for our analyses.

The association of stature (general: 2–17 years, and for each age cluster) with transversal anthropometric characteristics and body indices were analyzed using linear mixed-effects models (LMM). LMM were further used to analyze the association of bicondylar humerus diameter, bicondylar femur diameter and FI separately, with clinical and biochemical characteristics (covariates): age, parental height, age at diagnosis, (serum) levels of Hb, calcium, phosphate, PTH, ALP, and eGFR. Various covariate structure models were examined for LMM analyses, and the optimal model was chosen based on information criteria specific to each analysis and parameter group.

To emphasize disharmonies in body proportion, the extreme *z* score values of the anthropometric characteristics provided in Table 3 and Fig. 2 were subtracted, e.g., the lowest *z* score value was subtracted from the highest *z* score of considered anthropometric characteristic, in the given age group.

In graphic representation, LMM were employed to calculate predicted values from patients’ repeated measurements, accounting for the patient’s age while excluding covariate adjustment for repeated measurements (Fig. 2). A significance level of *p* < 0.05 was used to assess statistical significance. Data analysis and generated graphs were conducted using the standard statistical package SPSS for Windows, version 28.0 (IBM Corporation, NY, USA) and by use of GraphPad Prism 9.3.1 (GraphPad Software, Inc., San Diego, CA, USA).

## Results

### Patient characteristics

Clinical and biochemical characteristics of 109 children with XLH, all on supplementation therapy, are given in Table 1. Most patients were female (63.3%; *p* < 0.01). In 57.8% of patients, one parent was affected with XLH, of which 76.2% were the mothers (*p* < 0.01; Table 1). Median age at diagnosis and start of supplementation treatment was 2.00 years (IQR 0.06, 3.16) and 2.03 years (IQR 0.62, 3.42), respectively. The height of patients’ father and mother (Table 1) was significantly lower than the expected mean of the German adult healthy population (178.0 cm for men and 162.0 cm for women; *p* < 0.05). The mean weight related dosage of phosphate supplements in the whole study population was 50.2 mg/kg per day, based on elemental phosphorus and the mean calcitriol dosage was 25.6 ng/kg per day (Table 1). Both phosphate and calcitriol dosages were significantly lower in adolescent patients compared to children (each *p* < 0.01; Table 2).

**Table 2:** Clinical and biochemical characteristics of 109 children with XLH divided into three age groups, gathered through repeated measurements (estimated marginal means), with additionally conducted pairwise comparisons among age groups evaluated with the LMM

*Age group*	*Parameters*	*Estimated marginal mean (95% CI)*	*N measurements*
***A (2–6 years)***	Mean age, years	4.93 (4.63 to 5.22) B**, C**	109 of 109
	Serum hemoglobin, g/dL	12.5 (12.1 to 12.9) B**, C**	29 of 109
	Serum phosphate, *z* score	− 2.78 (− 3.18 to − 2.38) C*	80 of 109
	Serum calcium, *z* score	− 0.21 (− 0.45 to 0.03)	77 of 109
	Serum ALP, *z* score	2.61 (2.20 to 3.04)	79 of 109
	Serum PTH, *z* score	1.43 (1.02 to 1.84) C**	78 of 109
	Serum creatinine, µmol/l	30.9 (28.4 to 33.4) B**, C**	81 of 109
	eGFR, ml/min per 1.73 m^2^	130 (123 to 137) B*, C**	81 of 109
	Phosphate dosage, mg/kg/day	53.9 (5.3 to 102.7) C**	109 of 109
	Calcitriol dosage, ng/kg/day	28.4 (17.2 to 39.7) C*	109 of 109
***B (7–12 years)***	Mean age, years	10.01 (9.78 to 10.24) A**, C**	180 of 180
	Serum hemoglobin, g/dL	13.3 (13.0 to 13.5) A**, C**	81 of 180
	Serum phosphate, *z* score	− 3.13 (− 3.47 to − 2.79)	137 of 180
	Serum calcium, *z* score	− 0.25 (− 0.44 to − 0.05)	132 of 180
	Serum ALP, *z* score	2.36 (1.99 to 2.73) C**	132 of 180
	Serum PTH, *z* score	1.75 (1.41 to 2.09) C**	127 of 180
	Serum creatinine, µmol/l	41.0 (39.0 to 43.1) A**, C**	135 of 180
	eGFR, ml/min per 1.73 m^2^	121 (115 to 127) A*, C*	135 of 180
	Phosphate dosage, mg/kg/day	50.4 (1.7 to 99.1) C*	180 of 180
	Calcitriol dosage, ng/kg/day	25.6 (14.5 to 36.8) C*	180 of 180
***C (13–17 years)***	Mean age, years	15.41 (15.11 to 15.71) A**, B**	104 of 104
	Serum hemoglobin, g/dL	13.8 (13.5 to 14.1) A**, B**	51 of 104
	Serum phosphate, *z* score	− 3.42 (− 3.85 to − 3.00) A*	69 of 104
	Serum calcium, *z* score	− 0.15 (− 0.40 to − 0.10)	70 of 104
	Serum ALP, *z* score	3.06 (2.62 to 3.51) B**	65 of 104
	Serum PTH, *z* score	2.61 (2.17 to 3.05) A**, B**	66 of 104
	Serum creatinine, µmol/l	50.5 (47.8 to 53.1) A**, B**	69 of 104
	eGFR, ml/min per 1.73 m^2^	112 (104 to 119) A**, B*	69 of 104
	Phosphate dosage, mg/kg/day	46.3 (1.5 to 95.1) A**, B*	104 of 104
	Calcitriol dosage, ng/kg/day	22.7 (11.4 to 34.0) A*, B*	104 of 104

*N* describes number of valid measurements of valid yearly measurements

Statistically significant differences between the age groups were analyzed by pairwise comparison in respect of the significance level of (*p < 0.05) and (** *p* < 0.01)

*95% CI*, 95% confidence interval; *ALP*, alkaline phosphatase; *PTH*, parathyroid hormone; *eGFR*, estimated glomerular filtration rate

Estimated marginal mean (EMM) of serum phosphate *z*score was reduced (− 3.09 (95% CI − 3.39 to − 2.79)), while PTH *z* score (1.85 (95% CI: 1.55 to 2.15)) and ALP *z* score (2.60 (95% CI 2.26 to 2.94)) were markedly elevated. Conversely serum calcium *z* score (− 0.21 (95% CI: − 0.37 to − 0.06)), Hb (13.3 g/dL (95% CI: 13.08 to 13.51)), and eGFR (121 ml/min per 1.73 m^2^ (95% CI 116 to 126)) were found to be within the range of values observed in healthy children.

To analyze age-related changes in biochemical characteristics, patients were clustered into three age groups (A: 2–6 years, B: 7–12 years and C: 13–17 years; Table 2). Standardized phosphate decreased with age, resulting in a significant reduction from the youngest (A) to the oldest (C) age group (− 2.78 vs. − 3.42 *z* score; *p* < 0.05, respectively), while calcium *z* scores tended to be highest in adolescents (Table 2). ALP *z* scores were markedly elevated among all three age groups, with higher levels in adolescence (group C) compared to lowest levels in children aged 7–12 years (*p* < 0.05). On the other hand, PTH *z*scores show tendency of continuous cumulative increment across age groups that became significant with the start of adolescence (each *p* < 0.01 vs. groups A and B), and the prevalence of hyperparathyroidism increased steadily with age, reaching 14.3% in young children (group A), 27.3% in older children (group B), and 68.2% in adolescence (each *p* < 0.01; group C vs. groups A and B). As expected, serum creatinine levels and hemoglobin levels increased with age. Kidney function was normal in all patients (eGFR > 90 ml/min per 1.73 m^2^), although mean eGFR decreased significantly with age (A: 129, B: 120 vs. C: 111; each *p* < 0.05; Table 2). The prevalence of nephrocalcinosis was higher in adolescents than in children: 9.5% in group A (2–6 years), 11.36% in group B (7–12 years), and 40.9% in group C (13–17 years).

### Age-related changes in anthropometric parameters

**Fig. 1:**
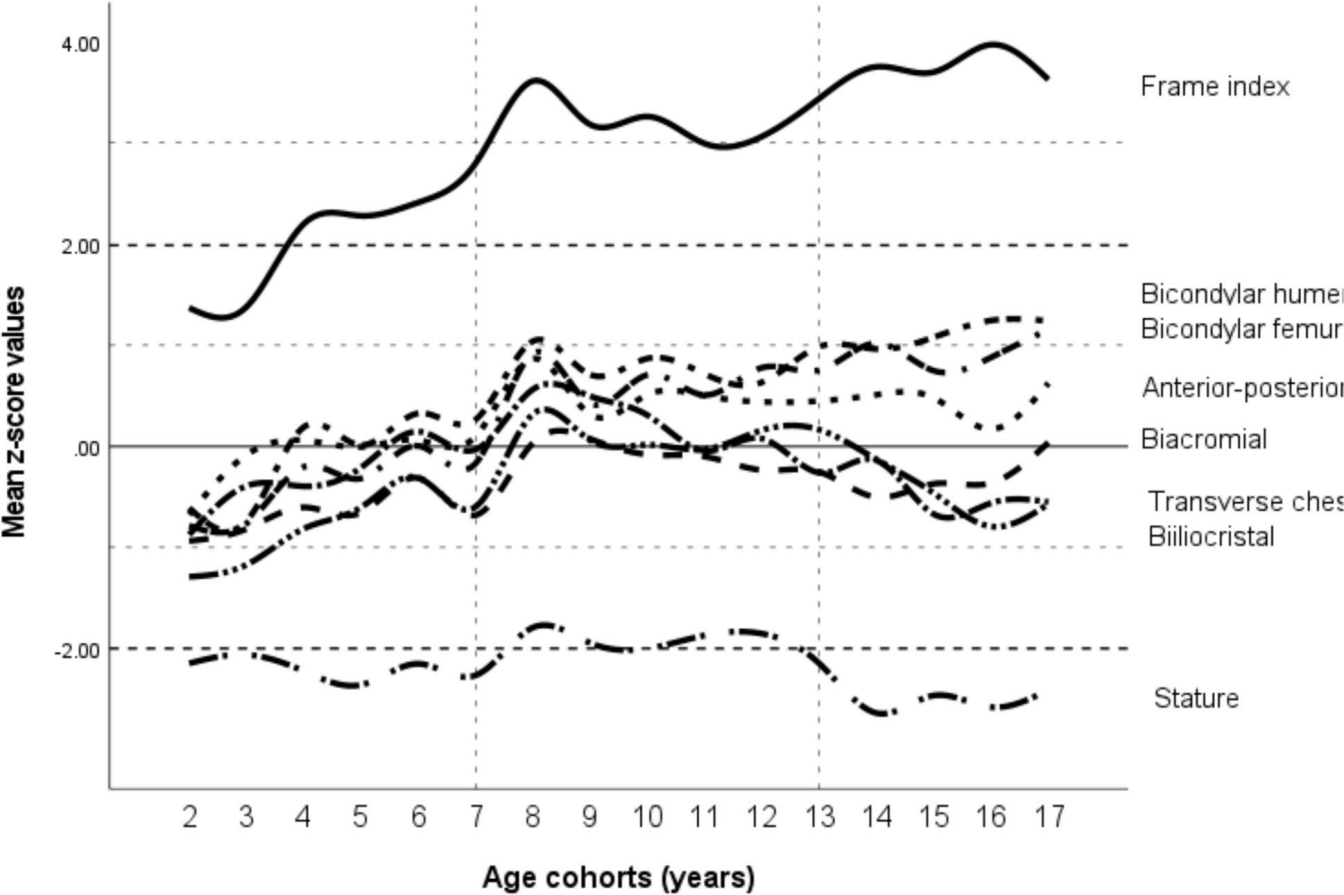
Mean *z* scores of transversal body diameters (bicondylar humerus, bicondylar femur, anterior–posterior chest, biacromial, transverse chest, biiliocristal diameter) as well as frame index and stature across the observed age range in 109 children with XLH. Dotted lines are for illustrative purposes only

**Fig. 2:**
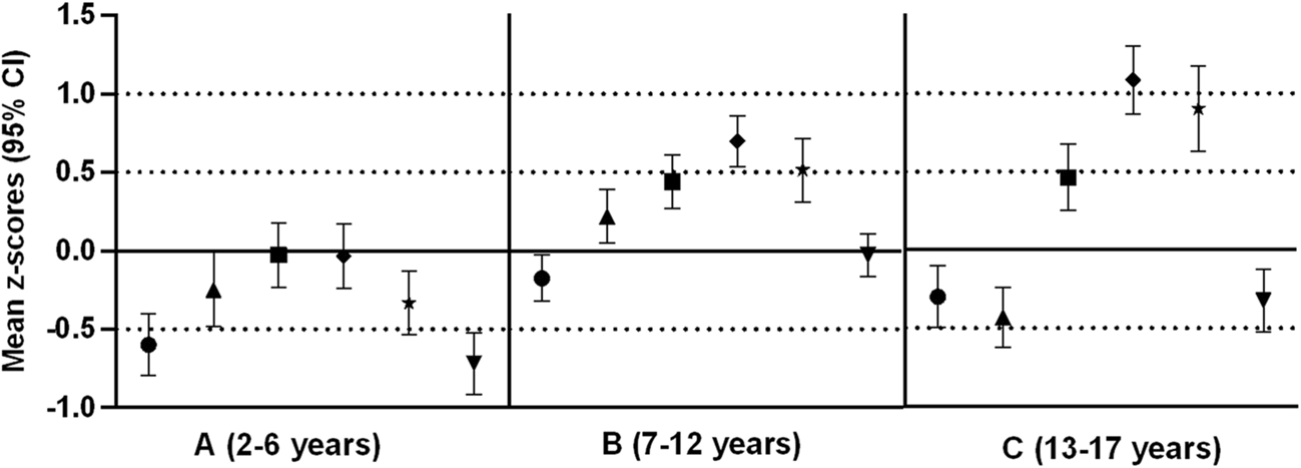
Mean *z* scores of transversal body diameters (biacromial, transverse chest, anterior–posterior chest, bicondylar humerus, bicondylar femur, biiliocristal diameter) clustered into three age groups of 109 children with XLH. Data are presented for the three age cohorts (2–6, 7–12, and 13–17 years) as age- and sex-dependent *z*scores. Error bars represent 95% confidence interval. Dotted lines are for illustrative purposes only, representing changes in patterns of transversal morphology across the three observed age groups

In general, body disproportion increased with age, despite continuous supplementation treatment, indicating desynchronized segmental growth (i.e., asynchronous growth of body segments such as trunk and limbs; Table 3; Figs. 1 and 2). At age two, when body disharmony (i.e., visible imbalance in body proportions) was least evident, stature exhibited the most reduction among all observed anthropometric characteristics (− 2.15 *z* score; Fig. 1), slightly improved by the age of 12 years (− 1.96 *z* score), followed by significant decrease to − 2.44 *z* score in adolescence (*p* < 0.01; Table 3), where physical disharmony was most pronounced. All transversal body characteristics were diminished in early childhood, but showed a synchronized consistent upward trend until the age of 8 years. At this age, only stature was significantly reduced compared to healthy children (Fig. 1). After the age of 8 years, anterior–posterior chest diameter constantly increased, while transverse chest diameter and biacromial diameter strongly decreased until adulthood (Fig. 1). At pre-pubertal age, all physical characteristics reached a stable state until progressive unbalanced growth of the limbs in relation to the trunk was observed at the age of 12 years (Fig. 1). This desynchronized segmental growth was characterized by significant increases of bicondylar width *z* scores of the extremities and consequently of the FI (each *p* < 0.05; Table 3) and simultaneously decreases of transverse chest and biiliocristal diameters (Fig. 1). In children with XLH advancing, desynchronized segmental growth resulted in increasing body disharmony (Fig. 1). Growth disparity patterns between body height and bicondylar humerus breadth were best expressed by the FI, as it displays the ratio of elbow width to stature.

**Table 3:** Anthropometric characteristics of 109 children with XLH, gathered through repeated measurements (estimated marginal means) during the observation period, and organized into three age groups (the characteristics are based on actual measurements evaluated with the LMM and completed by pairwise comparisons among age groups)

*Age group*	*A (2–6 years)*	*B (7–12 years)*	*C (13–17 years)*
***Number of patients [measurements]***	*21 [109]*	*44 [180]*	*44 [104]*
***Measurements***^a^	***Estimated marginal mean (95% CI)***	***Estimated marginal mean (95% CI)***	***Estimated marginal mean (95% CI)***
Stature, *z* score	− 2.20 (− 2.44 to − 1.97)	− 1.96 (− 2.14 to − 1.78) C*	− 2.44 (− 2.68 to − 2.20) B*
Sitting height, *z* score	− 1.01 (− 1.21 to − 0.81)	− 0.78 (− 0.94 to − 0.63)	− 0.73 (− 0.94 to − 0.53)
Biacromial diameter, *z* score	− 0.60 (− 0.79 to − 0.41) B**, C*	− 0.18 (− 0.32 to − 0.03) A**	− 0.30 (− 0.49 to − 0.11) A*
Transverse chest diameter, *z* score	− 0.25 (− 0.46 to − 0.04) B**	− 0.22 (− 0.05 to − 0.39) A**, C**	− 0.43 (− 0.65 to − 0.21) B**
Anterior–posterior chest diameter, *z* score	− 0.03 (− 0.24 to 0.19) B**, C*	0.44 (0.28 to 0.61) A**	0.46 (0.25 to 0.68) A*
Biiliocristal diameter, *z* score	− 0.72 (− 0.90 to − 0.53) B**, C*	− 0.03 (− 0.17 to 0.12) A**, C*	− 0.32 (− 0.51 to − 0.14) A*, B*
Bicondylar humerus diameter, *z* score	− 0.04 (− 0.24 to 0.17) B**, C**	0.70 (0.54 to 0.86) A**, C*	1.09 (0.88 to 1.30) A**, B*
Bicondylar femur diameter, *z* score	− 0.33 (− 0.58 to − 0.09) B**, C**	0.51 (0.32 to 0.71) A**, C*	0.90 (0.65 to 1.15) A**, B*
BMI, *z* score	0.96 (0.69 to 1.24) C**	0.99 (0.77 to 1.20) C**	1.63 (1.35 to 1.90) A**, B**
Sitting height index, *z* score	2.22 (1.96 to 2.48) B*, C*	2.63 (2.42 to 2.83) A*	2.69 (2.42 to 2.96) A*
Frame index, *z* score	2.08 (1.73 to 2.44) B*, C**	3.14 (2.87 to 3.42) A**, C*	3.68 (3.32 to 4.05) A**, B*

Statistically significant differences between the age groups were analyzed by pairwise comparison in respect of the significance level of (* p < 0.05) and (**p < 0.01)

95% CI, 95% confidence interval; BMI, body mass index

^a^Repeated measurement (estimated marginal means) during the observation period are based on actual measurement or annual average values and repeated measurements within the same individual (evaluated with the LMM, random patients, and age cohorts)

Figure 2 illustrates the age-related changes in the pattern of bodily dimensions in the three age groups. As an outcome of synchronized growth, from early childhood to pre-pubertal age, all transversal parameters increased significantly (each *p* < 0.05; Table 3; Fig. 2) and showed the same morphological pattern in these two age groups (A and B), with highest *z* scores in bicondylar humerus and anterior–posterior chest diameter, and lowest in biacromial and biiliocristal diameter. The pattern of transverse morphology then changed markedly in age group C (Fig. 2). To highlight disharmony in body proportion, ranges between the two extreme *z* score pairs of the anthropometric values in Fig. 2 were analyzed (difference between highest and lowest *z* score): in group A, the range was less distinct with a *z* score difference of 0.69 (anterior–posterior chest vs. biiliocristal diameter* z* scores; − 0.03 vs. − 0.72) compared to group B (0.88 *z* score difference; biacromial vs. bicondylar humerus diameter) and uppermost in group C (1.52 *z* score difference; bicondylar humerus vs. transversal chest diameter). Finally, the BMI *z* score was significantly higher in adolescents compared to younger children (*p* < 0.01; Table 3).

### Associations between stature and relevant transversal body characteristics, body mass index, sitting height index, and frame index

The associations of transversal body characteristics, BMI, SHI, and FI with stature varied by age (Table 4). Bicondylar humerus diameter and FI were generally associated with stature (independently of age), i.e., positively with bicondylar humerus, and negatively with FI (each age cluster *p* < 0.01). Wider bicondylar femur diameter was significantly associated with higher stature except in adolescents (Table 4). Interestingly, a significant negative association between biacromial diameter and stature was only observed in age group C (Table 4). In the entire patient cohort (2–17 years), stature was positively associated with transverse chest, anterior–posterior chest, biiliocristal diameter, bicondylar humerus, bicondylar femur diameter, and SHI (each *p* < 0.01) and negatively associated with BMI and FI (each *p* < 0.01; Table 4). Notably, only in the age group 2–6 years, higher BMI and lower SHI were significantly associated with stature (*p* < 0.01 and *p* < 0.05, respectively; Table 4). Finally, stature was positively associated with biiliocristal diameter at ages 13–17 years (*p* < 0.05).

**Table 4:** Associations between the stature and its predictors: relevant transversal body characteristics, body mass index, sitting height index and frame index in 109 children with XLH within complete observed period (2–17 years) and clustered into three age groups

	***Age group***			
	***All patients (2–17 years)***	***A (2–6 years)***	***B (7–12 years)***	***C (13–17 years)***
Intercept	− 0.39** (− 0.46 to − 0.32)	− 0.19** (− 0.29 to − 0.09)	− 0.35** (− 0.44 to − 0.27) 32)	− 0.32** (− 0.46 to − 1.19)
Biacromial diameter, *z* score	− 0.02 (− 0.07 to 0.03)	0.04 (− 0.03 to 0.12)	0.47 (0.01 to 0.11)	− 0.12* (− 0.20 to − 0.05)
Transverse chest diameter, *z* score	0.15** (0.07 to 0,16)	− 0.00 (− 0.06 to 0.05)	0.02 (− 0.04 to 0.07)	0.67 (− 0.02 to 0.16)
Anterior–posterior chest diameter, *z* score	0.04* (0.00 to 0.08)	− 0.01 (− 0.07 to 0.05)	− 0.02 (− 0.06 to 0.03)	0.02 (− 0.05 to 0.08)
Biiliocristal diameter, *z* score	0.10** (0.05 to 0.14)	− 0.06 (− 0.13 to 0.02)	0.03 (− 0.03 to 0.08)	0.07* (0.01 to 0.13)
Bicondylar humerus diameter, *z* score	0.96** (0.90 to 1.02)	1.09** (0.98 to 1.20)	0.96** (0.88 to 1.03)	1.23** (1.03 to 1.20)
Bicondylar femur diameter, *z* score	0.05* (0.00 to 0.09)	0.10* (− 0.02 to 0.18)	0.05* (0.00 to 0.10)	− 0.01 (− 0.07 to 0.05)
BMI, *z* score	− 0.11** (− 0.14 to − 0.07)	0.11** (0.05 to 0.17)	0.01 (− 0.05 to 0.06)	− 0.04 (− 0.11 to 0.02)
Sitting height index, *z* score	0.02* (− 0.01 to 0.05)	− 0.07* (− 0.12 to − 0.02)	− 0.02 (− 0.05 to − 0.01)	0.05 (− 0.00 to 0.10)
Frame index, *z* score	− 0.76** (− 0.80 to − 0.72)	− 0.91** (− 0.97 to − 0,85)	− 0.72** (− 0.76 to − 0.67)	− 0.92** (− 0.98 to − 0.85)

Data are presented as *β* values (95% confidence intervals)

Algebraic sign in *β* values expresses positive (+) or negative (–) isolated covariation between stature and its predictor

Association between the stature and each anthropometric predictor regarding significance level (*p < 0.05) and (**p < 0.01)

*BMI*, Body mass index

### Associations between physical characteristics (bicondylar humerus diameter, bicondylar femur diameter and frame index) and relevant clinical characteristics

As bicondylar humerus, femur diameter and FI were revealed to be the best predictors of stature in our XLH sample, we then investigated potential clinical predictors for these three characteristics using linear mixed-effects models (Table 5). Investigation of the separate age groups showed that the impact of predictors depends on the child’s age, as the number of statistically significant predictors was considerably higher in the youngest age group than in the older (Table 5). Interestingly, there were no statistically significant associations found between clinical characteristics within the age cluster 7–12 years. At the age of 2–6 years (group A), all observed clinical characteristics (except for parental height) were significantly associated with bicondylar femur diameter: positively with age, Hb, calcium and ALP, and negatively with age at diagnosis, phosphate, PTH, and eGFR (each *p* < 0.05). In addition, bicondylar humerus was significantly positively associated with eGFR in age group 2–6 years (*p* < 0.05). FI was significantly associated with all biochemical characteristics in group A; positive associations were observed with age, Hb, ALP, and eGFR and negative associations with calcium, phosphate, and PTH (each *p* < 0.01). In adolescence (13–17 years), bicondylar humerus diameter was positively associated with Hb and calcium (each *p* < 0.05). However, no statistically significant association was identified between bicondylar femur diameter and FI with the observed clinical determinants within the same period. In addition, no statistically significant association was found between parental height and the observed physical characteristics across all age clusters.

**Table 5:** Associations between target anthropometric characteristics (bicondylar humerus diameter, bicondylar femur diameter and frame index) and relevant clinical determinants in 109 children with XLH; in overall observed period (2–17 years) and clustered into three age groups (LMM)

	**Clinical parameters**	***All patients (2–17 years)***	***A (2–6 years)***	***B (7–12 years)***	***C (13–17 years)***
**Bicondylar humerus diameter**	Intercept	− 2.01 (− 5.86 to 1.84)	− 1.08 (− 10.05 to 7.88)	− 0.33 (− 4.80 to 4.15)	− 4.79 (− 12.62 to 3.04)
	Age, years	0.12** (− 0.08 to 0.15)	0.17 (− 0.07 to 0.41)	0.04 (− 0.04 to 0.13)	0.06 (− 0.15 to 0.27)
	Parental height, cm (× 1000)	0.01 (0.00 to 0.00)	− 0.01 (− 0.00 to 0.00)	0.02 (− 0.00 to 0.00)	0.04 (0.00 to 0.00)
	Age at diagnosis, years	0.02 (− 0.18 to 0.14)	0.15 (− 0.27 to 0.57)	0.12 (− 0.13 to 0.37)	− 0.03 (− 0.25 to 0.19)
	Hemoglobin, g/dL	0.7 (− 0.08 to 0.21)	− 0.13 (− 0.48 to 0.21)	− 0.04 (− 0.23 to 0.16)	0.33* (0.05 to 0.61)
	Calcium, *z* score	0.70 (− 0.05 to 0.18)	− 0.15 (− 0.36 to 0.06)	− 0.05 (− 0.20 to 0.09)	0.26* (− 0.05 to 0.46)
	Phosphate, *z* score	− 0.01 (− 0.10 to 0.08)	− 0.07 (− 0.33 to 0.20)	− 0.02 (− 0.12 to 0.08)	− 0.07 (− 0.24 to 0.11)
	ALP, *z* score	0.12** (0.04 to 0.20)	0.10 (− 0.12 to 0.32)	0.04 (− 0.11 to 0.19)	0.03 (− 0.13 to 0.19)
	PTH, *z* score	− 0.04 (− 0.14 to 0.05)	− 0.10 (− 0.33 to 0.12)	0.05 (− 0.06 to 0.15)	− 0.01 (− 0.21 to 0.24)
	eGFR, ml/min per 1.73 m^2^ (× 10)	0.01 (0.04 to 0.05)	1.20* (0.03 to 0.21)	0.00 (− 0.06 to 0.06)	− 0.08 (− 0.22 to 0.06)
**Bicondylar femur diameter**	Intercept	− 1.25 (− 5.86 to 3.36)	− 13.92** (− 21.00 to − 6.84)	− 2.60 (− 8.72 to 3.52)	− 1.86 (− 11.73 to 8.02)
	Age (× 10; 7–12)	0.10** (0.04 to 0.15)	1.04** (1.04 to 1.04)	0.02 (− 1.14 to 1.09)	0.04 (− 0.28 to 0.35)
	Parental height (× 1000)	0.07 (− 0.07 to 0.00)	0.00 (− 0.01 to 1.00)	0.08 (0.00 to 0.00)	0.00 (− 0.00 to 0.00)
	Age at diagnosis, years	− 0.07 (− 0.25 to 0.12)	− 0.76* (− 1.27 to − 0.24)	0.14 (− 0.21 to 0.48)	− 0.17 (− 0.40 to 0.07)
	Hemoglobin, g/dL	− 0.12 (− 0.33 to 0.08)	0.11** (0.11 to 0.11)	0.02 (− 0.25 to 0.29)	− 0.12 (− 0.50 to 0.26)
	Calcium, *z* score	− 0.03 (− 0.19 to 0.14)	0.42** (0.42 to 0.42)	0.15 (− 0.05 to 0.35)	− 0.09 (− 0.38 to 0.21)
	Phosphate, *z* score (× 10)	− 0.40 (− 1.59 to 0.78)	− 1.60** (− 1.62 to − 1.58)	0.02 (− 1.33 to 1.36)	− 1.27 (− 3.76 to 1.21)
	ALP, *z* score	0.13* (0.02 to 0.24)	0.62** (0.61 to 0.62)	0.03 (− 0.17 to 0.23)	0.21 (− 0.02 to 0.44)
	PTH, *z* score	0.11 (− 0.02 to 0.24)	− 0.32** (− 0.33 to − 0.32)	0.10 (− 0.04 to 0.24)	0.25 (− 0.03 to 0.53)
	eGFR, ml/min per 1.73 m^2^ (× 10)	0.00 (− 0.07 to 0.07)	− 0.03** (− 0.03 to − 0.03)	0.04 (− 0.04 to 0.12)	− 0.07 (− 0.26 to 0.13)
**Frame index**	Intercept	4.95 (− 1.44 to 11.33)	− 16.18** (− 26.35 to − 6.00)	6.65 (− 1.15 to 14.44)	5.32 (− 8.64 to 19.28)
	Age, years	0.15** (0.09 to 0.20)	0.61** (0.61 to 0.61)	− 0.03 (− 0.16 to 0.10)	− 0.17 (− 0.48 to 0.15)
	Parental height, cm (× 1000)	0.00 (0.00 to 0.08)	0.10 (−.00 to 0.00)	0.00 (− 0.00 to 0,06)	0.09 (0.00 to 0.00)
	Age at diagnosis, years (× 10)	0.40 (− 2.42 to 2.322)	0.04 (− 7.38 to 7.46)	3.15 (− 1.53 to 7.84)	− 0.46 (− 4.89 to 3.97)
	Hemoglobin g/dL	0.02 (− 0.19 to 0.23)	0.35** (0.35 to 0.36)	0.14 (− 0.16 to 0.45)	0.30 (− 0.15 to 0.75)
	Calcium, *z* score	− 0.03 (− 0.19 to 0.14)	− 0.04** (− 0.04 to − 0.03)	− 0.04 (− 0.26 to 0.19)	− 0.07 (− 0.40 to 0.25)
	Phosphate, *z* score	− 0.07 (− 0.20 to 0.06)	− 1.08** (− 1.08 to − 1.08)	− 0.03 (− 0.18 to 0.13)	− 0.11 (− 0.39 to 0.18)
	ALP, *z* score	0.05 (− 0.06 to 0.17)	0.59** (0.59 to 0.59)	− 0.07 (− 0.30 to 0.17)	0.08 (− 0.17 to 0.32)
	PTH, *z* score	− 0.02 (− 0.16 to 0.11)	− 0.53** (− 0.54 to − 0.53)	0.04 (− 0.11 to 0.19)	− 0.09 (− 0.47 to 0.30)
	eGFR, ml/min per 1.73 m^2^ (× 10)	0.03 (− 0.11 to 0.04)	0.25** (0.25 to 0.25)	− 0.02 (− 0.11 to 0.07)	− 0.06 (− 0.29 to 1.70)

Data are presented as *ß* values (95% confidence intervals)

Algebraic sign in *ß* values expresses positive (+) or negative (−) isolated association/covariation between criterion and predictor’s variable

Associations between target anthropometric characteristics and relevant clinical determinants regarding significance level (*p < 0.05) and (**p < 0.01)

*ALP*, Alkaline phosphatase; *PTH*, parathyroid hormone; *eGFR*, estimated glomerular filtration rate

## Discussion

This prospective, multicenter observational study, conducted over a 25-year period and including 109 children on supplementation therapy, provides new insights into age-related morphological changes in patients with XLH by investigating transversal body dimensions, body proportions, and their association with stature as well as their clinical determinants. Patients showed an unbalanced growth in transversal body dimensions with age, with ever increasing FI. The latter may be used in the future to monitor bone health in children with XLH in clinical practice or in trials, for example when comparing the efficacy of burosumab treatment with that of supplementation therapy.

In our XLH patient cohort, evaluation of transversal characteristics revealed more extended dynamics in this population compared to body height, as the impaired stature changed only slightly with increasing age with a difference of − 0.24 *z* score between early childhood and adolescence. As expected, XLH patients showed persisting impaired bone metabolism with mineralization defects, as indicated by markedly elevated ALP levels [5] (biochemical marker for rachitic activity, despite supplementation of phosphate and treatment with active vitamin D analogs at dosages recommended in current guidelines [4, 26]). This was associated with compensatory mechanisms to ensure functionality of the musculoskeletal system [27]. The latter was characterized by an increase in the mediolateral width of tubular bones, a hallmark of rickets in children. Observations from our study strongly support the principle of “form follows function”, which is in agreement with Wolff’s Law [28, 29], reflecting that skeletal structure adjusts to functional needs in growth due to metabolic abnormalities [30–32]. The manifestation of skeletal adaptation to rickets, as noted in the present study, is an increased FI. In healthy populations, bones with higher mineralization exhibit a slender appearance [30]. Markedly increased femoral mediolateral diameters and bone mass have previously been described in 11 children with XLH using magnetic resonance imaging (MRI) by Nguyen-Khac et al. [16]. Our study population presented with markedly elevated FI on account of XLH-specific pathomorphology (impaired stature and widened tubular bones). Consequently, FI increased significantly with age as tubular bone widening progressed and was further associated with impaired stature, irrespective of age (each *p* < 0.01). FI has already been used to investigate bone robustness (strength) in the context of bone remodeling [14, 21]. Furthermore, FI reveals an additional aspect of bone health, correlating with physical activity in healthy children; Rietsch et al. also noted reduced skeletal robustness in subjects with low physical activity [14] and Scheffler et al. noted bone robustness increases with hours of weekly sports activity [11]. Therefore, the findings of this study indicate that patients with XLH must achieve very high FI (> 2 *z* scores) to accomplish mobility and participation in physical activities, due to the limitations of their musculoskeletal system [27, 33].

Widening of tubular bones was significantly associated with the biochemical parameters of bone metabolism: increased ALP was significantly associated with wider bicondylar breadth of the limbs in all patients observed (2–17 years), and additionally increased FI with elevated ALP and moreover with reduced levels of phosphate and calcium in early childhood. During this phase of rapid growth at ages 2–6 years, the dynamics of body diameters were most synchronized and, interestingly, accompanied by highest phosphate levels and lowest PTH levels, compared to older children (Table 2). Notably, ALP was higher in group A compared to B, which is in agreement with normative data investigations by Turan et al., demonstrating ALP is elevated during rapid growth phases in healthy children, as 7–12 year old children presented with a decreased intensity of transversal morphological changes [26] (Fig. 1).

In the present study, associations between BMI and stature varied depending on age (Table 4). Elevated BMI was significantly associated with reduced height over the entire study cohort (2–17 years). However, in children aged 2–6 years, BMI was significantly associated with taller stature. A higher prevalence of obesity in adolescents with XLH (> 15 years) compared to younger children was observed by Zhukouskaya et al. [34], which is in agreement with the findings of this study showing that no child aged 2–6 years presented with a BMI above 24.2 kg/m^2^ and on the other hand 13% of adolescents presented with BMI ≥ 30 kg/m^2^. The elevated BMI present in the younger age groups therefore appears to be attributed more to body disproportion (impaired stature and greater proportional weight [35]) due to the underlying pathology than to actual overweight or obesity. In our XLH cohort, BMI exhibited a progressive increase with age and reached its most pronounced levels during adolescence, reflecting the limited physical functioning and physical activity observed among patients with XLH receiving supplementation treatment [8, 16, 18, 36–38]. Of note, in our study population, 21.1% of patients required lower limb surgery, of which adolescents presented with the highest prevalence (group A: 9.25%, group B: 13.64%, group C: 34.9%). Finally, decreased rates of muscular adenosine triphosphate synthesis, due to reduced phosphate concentrations in blood serum levels, have been observed in mice with hypophosphatemia, which may contribute to muscle weakness in our patient cohort [38]. Furthermore, in this study’s adolescent patients with XLH, the biacromial diameter, which is wider in athletic individuals (correlation with musculoskeletal fitness [8]), was significantly associated with an impaired stature, which indicates the presence of skeletal adaptation to impaired fitness (Table 4).

Notably, the dynamics of the trunk segmental growth generally align with the dynamics of all transversal parameters observed at ages 2–6 years, then reached a steady state after the physiological age of complementation of lung development was attained and then decreased during adolescence. Transverse chest diameter may be most reflective of skeletal ribcage growth, affected by the development of the lungs; hence, this peaks at the age of 8 years, coinciding with completion of lung development [39]. Pelvic size varies independent of body height, as described by Veena et al. [9]. In our study, biiliocristal diameter expanded during ages 12–13 years in preparation for sexual maturity, which agrees with Stuart and Veena’s observation that rising estrogen levels lead to expansion of the pelvis in females [9]. This peak coincided with the age of onset of menarche, which is strongly associated with pelvic expansion in female adolescents [10]. We noted significant positive associations between biiliocristal diameter and stature in adolescence, indicating that patients with XLH with wider pelvic diameters, considered as optimized maturity, generally grow better in height (Table 4).

The study population showed normal kidney function (Tables 1 and 2). However, the observed significant age-related decline in eGFR may be attributed to complications of conventional treatment [1, 40], e.g., nephrocalcinosis, which was noted in 22.9% of our patients. Notably, the prevalence of nephrocalcinosis in our population increased with age and duration of supplementation and was highest in group C (13–17 years) at 40.9%. Interestingly, phosphate *z* scores decreased significantly with age, which can be explained, at least in part, by the significantly lower weight-based dosages of phosphate supplements and calcitriol in adolescent patients compared to children (Table 2). The latter may have been a response to the increasing incidence of nephrocalcinosis with age, possibly requiring a dose reduction by treating physicians [1]. Additionally, XLH patients exhibit hyperparathyroidism, which is a known complication in patients with XLH receiving supplemental treatment and becomes more pronounced with increasing therapy duration [40]. Among the various age groups examined in this study, adolescents exhibited the significantly highest prevalence of hyperparathyroidism at 68.2% (A: 14.3%, B: 27.3%; *p* < 0.01).

The most significant associations of transversal dimensions and FI with clinical and biochemical predictors were observed in children aged 2–6 years, indicating a higher susceptibility to changes in bone metabolism in young patients with XLH. Therefore, our findings emphasize the importance of early treatment initiation for proper transversal growth, consistent with previous research, showing that early treatment leads to greater improvements in body height and mitigated rickets [41]. The observed biochemical predictors, which were associated with FI in our findings, show rapid improvements under burosumab treatment [1, 42]. Therefore, FI appears to be a useful measure of treatment efficacy in XLH patients under burosumab treatment and when switched from supplementation therapy to burosumab. The economic viability (low costs) and broad applicability of FI render it a suitable tool for monitoring bone health in children with XLH. Moreover, in comparison with alternative measurement methodologies, such as radiography (X-rays), the assessment of anthropometric measurements (e.g., bicondylar humerus and femur diameter) offers the benefit of being devoid of any side effects associated with irradiation (particularly in view of repeated measurements) and can be utilized without the necessity of employing radiographic apparatus.

In conclusion, our findings provide valuable insights into the dynamics of transversal disproportional growth in XLH. Acquiring better understanding of these dynamics can help develop more effective strategies to prevent and mitigate body disproportion in patients with skeletal diseases, such as XLH. This can be achieved through improved timing and monitoring of therapies by utilizing the frame index.

## Supplementary Information

Below is the link to the electronic supplementary material.

Graphical abstract (PPTX 108 KB)

## Data Availability

The data supporting the findings of this study are available on request from the corresponding author. Data are not publicly available due to privacy or ethical restrictions.
