# Supplementary material for: Growth dynamics of transversal body dimensions and proportions, with related clinical determinants in children with X-linked hypophosphatemia treated with phosphate supplements and active vitamin D
Source: Pediatr Nephrol. 2025 Jun 10;40(10):3187–200. doi: 10.1007/s00467-025-06841-y (PMC12401769; doi:10.1007/s00467-025-06841-y)
Supplement: Supplementary file 1 — Graphical abstract (PPTX 108 KB) [file 467_2025_6841_MOESM1_ESM.pptx]

## Slide 1
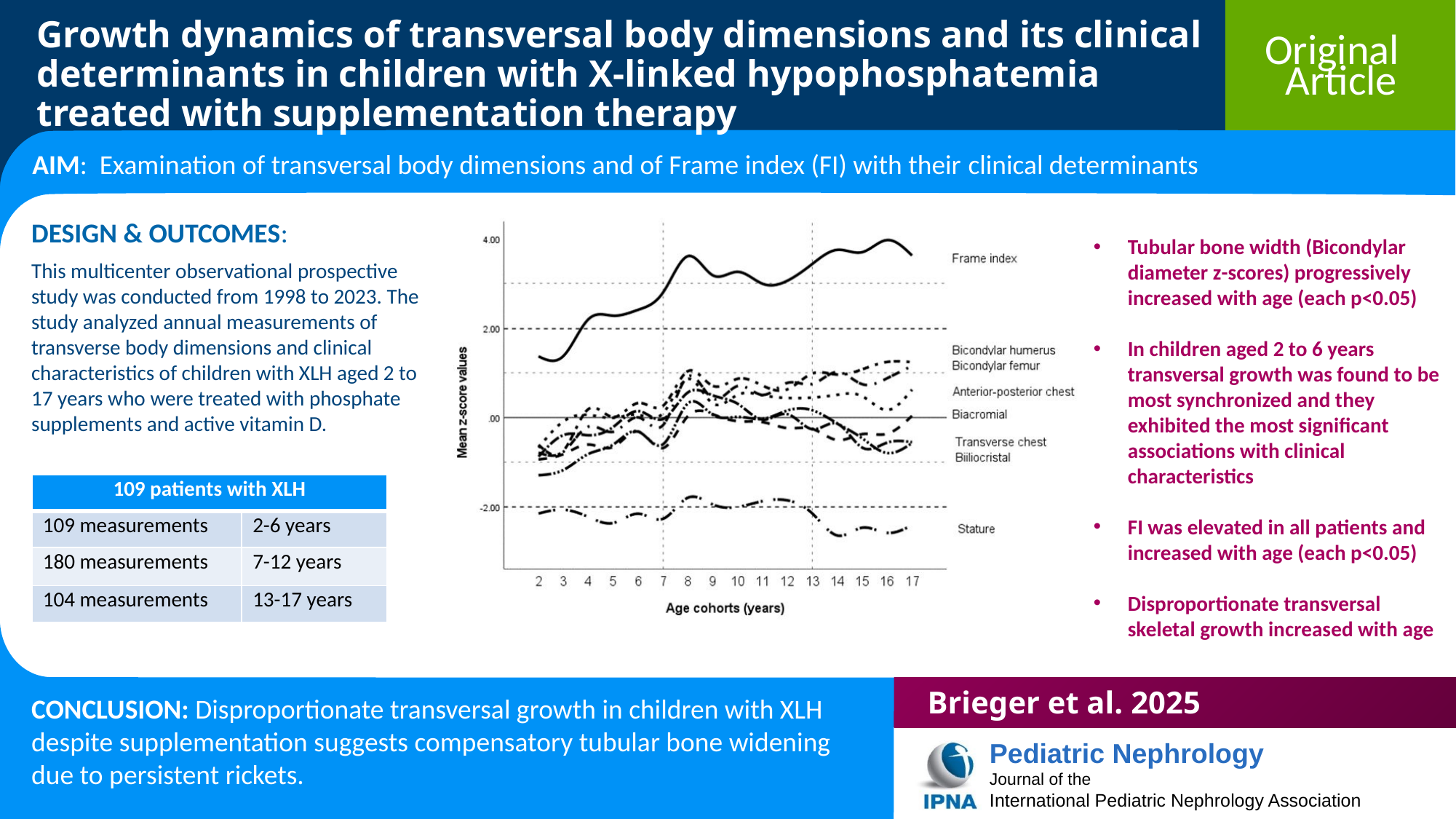

Growth dynamics of transversal body dimensions and its clinical determinants in children with X-linked hypophosphatemia treated with supplementation therapy
AIM: Examination of transversal body dimensions and of Frame index (FI) with their clinical determinants
Tubular bone width (Bicondylar diameter z-scores) progressively increased with age (each p<0.05)
In children aged 2 to 6 years transversal growth was found to be most synchronized and they exhibited the most significant associations with clinical characteristics
FI was elevated in all patients and increased with age (each p<0.05)
Disproportionate transversal skeletal growth increased with age
DESIGN & OUTCOMES:
This multicenter observational prospective study was conducted from 1998 to 2023. The study analyzed annual measurements of transverse body dimensions and clinical characteristics of children with XLH aged 2 to 17 years who were treated with phosphate supplements and active vitamin D.
| 109 patients with XLH | |
| --- | --- |
| 109 measurements | 2-6 years |
| 180 measurements | 7-12 years |
| 104 measurements | 13-17 years |
Brieger et al. 2025
CONCLUSION: Disproportionate transversal growth in children with XLH despite supplementation suggests compensatory tubular bone widening due to persistent rickets.
